# Xanthatin inhibits STAT3 and NF‐κB signalling by covalently binding to JAK and IKK kinases

**DOI:** 10.1111/jcmm.14322

**Published:** 2019-04-16

**Authors:** Man Liu, Cheng‐qian Xiao, Ming‐wei Sun, Min‐jia Tan, Li‐hong Hu, Qiang Yu

**Affiliations:** ^1^ Shanghai Institute of Materia Medica, Chinese Academy of Sciences Shanghai PR China; ^2^ University of Chinese Academy of Sciences Beijing PR China; ^3^ Jiangsu Key Laboratory for Functional Substance of Chinese Medicine, Jiangsu Collaborative Innovation Center of Chinese Medicinal Resources Industrialization, Stake Key Laboratory Cultivation Base for TCM Quality and Efficacy, School of Pharmacy Nanjing University of Chinese Medicine Nanjing PR China; ^4^ State Key Laboratory of Drug Research Shanghai Institute of Materia Medica, Chinese Academy of Sciences Shanghai PR China; ^5^ The Chemical Proteomics Center Shanghai Institute of Materia Medica, Chinese Academy of Sciences Shanghai PR China

**Keywords:** covalent interaction, IKK inhibitor, JAK inhibitor, natural compound

## Abstract

Aberrant activation of the signal transducer and activator of transcription 3 (STAT3) and the nuclear factor‐κB (NF‐κB) signalling pathways is associated with the development of cancer and inflammatory diseases. JAKs and IKKs are the key regulators in the STAT3 and NF‐κB signalling respectively. Therefore, the two families of kinases have been the major targets for developing drugs to regulate the two signalling pathways. Here, we report a natural compound xanthatin from the traditional Chinese medicinal herb *Xanthium L*. as a potent inhibitor of both STAT3 and NF‐κB signalling pathways. Our data demonstrated that xanthatin was a covalent inhibitor and its activities depended on its α‐methylene‐γ‐butyrolactone group. It preferentially interacted with the Cys243 of JAK2 and the Cys412 and Cys464 of IKKβ to inactivate their activities. In doing so, xanthatin preferentially inhibited the growth of cancer cell lines that have constitutively activated STAT3 and p65. These data suggest that xanthatin may be a promising anticancer and anti‐inflammation drug candidate.

## 1 INTRODUCTION

The Janus kinase (JAK)/signal transducer and activator of transcription (STAT) signalling pathways play important roles in various biological processes, including immune responses, inflammation, hematopoiesis and oncogenesis by regulating cell growth, survival and differentiation.^1^, ^2^ In mammalian cells, the JAK family consists of four members: JAK1, JAK2, JAK3 and Tyk2. Ligand‐induced receptor oligomerization leads to transphosphorylation and activation of JAKs and phosphorylation of the receptors and their substrates STATs. Phosphorylated STATs are then dimerized and translocate to the nucleus and function as transcription factors.^2^, ^4^, ^5^ Accumulating evidence indicate that abnormalities in the JAK/STAT signalling pathways are associated with inflammation and cancer.^7^, ^8^ JAKs are the key enzymes in regulating these pathways and are promising drug targets. A pan‐JAK inhibitor ruxolitinib has already been approved by FDA for the treatment of myeloproliferative neoplasms.^17^ A JAK3 inhibitor tofacitinib has also been approved for the treatment of rheumatoid arthritis and is being evaluated for the treatment of psoriasis and inflammatory bowel disease.^18^, ^19^

The transcription factor nuclear factor‐κB (NF‐κB) regulates the expression of a wide range of genes vital for immune responses and cell survival and proliferation.^20^ In unstimulated cells, the NF‐κB dimers are sequestered in the cytoplasm by inhibitory subunit IκBs (Inhibitor of κB). In the canonical NF‐κB pathway, an IKK (IκB kinase) complex composed of IKKα, IKKβ and IKKγ regulates the activation of NF‐κB. Upon stimulation, the IKK complex phosphorylates IκBα, leading to the degradation of IκBα and the release of NF‐κB complex. NF‐κB can be further phosphorylated by IKK complex, resulting in enhanced transcriptional activity.^21^, ^22^ Constitutive NF‐κB activation occurs in chronic inflammation and in a wide range of haematological and solid tumours, making NF‐κB signalling pathway an attractive target for the development of anti‐inflammatory and anticancer drugs.^25^, ^26^ Because of the indispensable role of IKK in the activation of NF‐κB signalling pathway, development of IKK inhibitors is an effective approach to block NF‐κB signalling.^31^, ^32^

Xanthatin (Xa) is a bioactive compound identified from the plant *Xanthium L.*, which has been used as an anti‐inflammatory herb in traditional Chinese medicine to treat diseases such as nasal sinusitis and arthritis. Xanthatin has been reported to inhibit both signal transducer and activator of transcription 3 (STAT3) and NF‐κB activation, but the molecular mechanisms of action are still unknown.^34^, ^35^

Here, we report that xanthatin is a covalent and selective inhibitor of JAKs and IKKs. It preferred JAKs and IKKs over abundant proteins, such as tubulin and actin and had no effects on many other kinases. Moreover, xanthatin selectively inhibited the growth of cancer cells with constitutively activated STAT3. These data explain how *Xanthium L.* plant may act as an anti‐inflammatory herb and suggest that xanthatin may be a promising anti‐inflammation and anticancer drug candidate.

## 2 MATERIALS AND METHODS

### 2.1 The preparation of Xa, Xa‐2 and Xa‐3

The xanthatin (Xa), 11α, 13‐dihydroxanthatin (Xa‐2) and xanthinosin (Xa‐3) were isolated from the aerial parts of *Xanthium mogolium Kitag,* as described previously.^37^ Their structures were determined by LC‐MS, ^1^H NMR and ^13^C NMR and are in accordance with the published data.

### 2.2 The preparation of Xa‐1

Xanthatin (100 mg, 0.41 mmol) was dissolved in methanol (10 mL), followed by addition of Pd/C (500 mg) under N_2_ protection. H_2_ (~1 atm) was then filled into the flask and the mixture was incubated with stirring for 1 hour at room temperature. The mixture was then filtrated with celite and the filtrate was concentrated under reduced pressure. The concentrated residue was purified with silica chromatograph to yield the colourless oil Xa‐1 quantitatively.

### 2.3 The preparation of Alk‐Xa

Five hundred milligram Xanthatin (2.03 mmol) was dissolved in 1,4‐dioxane/H_2_O (15 mL/8 mL) and was incubated in ice‐water bath with stirring. Eight millilitre 5.2% sodium hypochlorite was added by dropping and incubated for 3 hours with stirring. About 1.62 *g* KI (6.09 mmol) and 21 mL saturated NaHSO_3_ aqueous solution were then added sequentially by dropping until the solution turned yellow. Then 3 N HCl was added to adjust the pH to ~2. After stirring overnight at room temperature, xanthanic acid was crystallized and dried as white solid crystals with a yield of 70%.

Fifty milligram xanthanic acid (0.20 mmol) was dissolved in 5 mL anhydrous dichloromethane (DCM). About 0.23 mL (2.72 mmol) oxalyl chloride was added under N_2_ protection, followed by addition of 1 drop of catalytic *N*, *N* ‐dimethylformamide. The reaction was then transferred to a 50°C oil bath and stirred for 2 hours. Then the solvent and the redundant oxalyl chloride were removed under reduced pressure. The residue was dissolved in 3 mL anhydrous DCM and was stirred in ice‐water bath under N_2_ protection, followed by addition of 13 μL 2‐propynylamine (0.20 mmol) and 55 μL triethylamine (0.40 mmol). Thirty minutes later, the mixture was diluted with 40 mL DCM and washed with 30 mL saturated NaHCO_3_ aqueous solution and 30 mL saturated NaCl aqueous solution sequentially and dried with anhydrous Na_2_SO_4_. The organic solvent was removed under reduced pressure and the remaining residue was purified with silica gel chromatograph to produce 38 mg Alk‐Xa with a yield of 67%.

### 2.4 Cell culture and reagents

HEK293/NF‐κB and HepG2/STAT3 cells are gifts from Professor Xin‐Yuan Fu (National University of Singapore, Singapore), which were HEK293 and HepG2 cells stably transfected with NF‐κB‐responsive and STAT3‐responsive firefly luciferase reporter plasmids respectively. All other cell lines were obtained from the American Type Culture Collection. HEK293/NF‐κB, HEK293, MEF, T47D, BT474, MCF‐7, Hs578t, MDA‐MB‐453 cells were cultured in DMEM (Gibco, Grand Island, NY, USA) supplemented with 10% (v/v) FBS (Gibco, Grand Island, NY, USA). MDA‐MB‐231 and MDA‐MB‐468 cells were cultured in RPMI 1640 medium (Gibco, Grand Island, NY, USA) supplemented with 10% FBS. HepG2/STAT3 cells were cultured in Minimum Essential Medium Alpha medium (Gibco, Grand Island, NY, USA) supplemented with 10% (v/v) FBS. All cell lines were cultured at 37°C in a humidified atmosphere of 95% air and 5% CO_2_.

AZD1480 was purchased from Selleck (Shanghai, China). DTT and MTT were purchased from Genebase (Shanghai, China). GSH was obtained from Shanghai Sibas Bioscience (Shanghai, China). IL‐6 and IFNα were purchased from Peprotech (Saint Paul, MN, USA), TNFα was purchased from R&D Systems (Minneapolis, MN, USA) and Biotin‐azide was purchased from Cayman chemical (#13040). Streptavidin agarose was purchased from Thermo Fisher Scientific (#20357). Rhodamine‐azide (#760765), Tris (3‐hydroxypropyltriazolylmethyl) amine (THPTA, #762342), sodium ascorbate (#A4034) and copper sulphate (#451657) were purchased from Sigma‐Aldrich (Saint Louis, MO, USA).

The plasmid encoding the JAK2‐FLAG was a gift from Prof. David E. Levy (New York University). The plasmid encoding the IKKβ‐FLAG was a gift from Prof. Tom Gilmore (Boston University).

### 2.5 Antibodies

The following antibodies were purchased from Cell Signaling Technology (Boston, MA, USA): phospho‐EGFR (#3777), EGFR (#4267), phospho‐InsR/IGF1R (#3024), IGF1R (#3018), phospho‐Y705‐STAT3 (# 9145), STAT3 (#9139), STAT1 (#9172), phospho‐ Y1007/1008‐JAK2 (#3776), JAK2 (#3230), phospho‐ Y1022/1023‐JAK1 ( #3331), JAK1 (#3332), phospho‐ Y1054/1055‐TYK2 (#9321), TYK2 (#9312), phospho‐ S32‐Ikbα (#2859), Ikbα (#4814), phospho‐S176/180‐IKKα/β (#2697), IKKα (#2682), IKKβ (#8943), NF‐κB p65(#8242), phospho‐S536‐NF‐κB p65(#3033). The antibodies for gp130 (#sc‐656) and α‐Tubulin (#SC‐5286) were purchased from Santa Cruz Biotechnology (Dallas, TX, USA). The antibody for β‐Actin was purchased from Abmart (#P30002M). The antibody for phospho‐Y1230/1231‐VEGFR3 (#CY1115) was purchased from Cell Applications. Secondary HRP‐conjugated antibodies were purchased from Multi Sciences Biotech (Hangzhou, China). The anti‐Flag affinity gel was purchased from Bimake (#B23101, Shanghai, China).

### 2.6 Luciferase assays

HEK293/NF‐κB or HepG2/STAT3 cells were seeded into 96‐well cell culture plates and cultured to 90% confluence. Cells were then treated with xanthatin for 1 hour followed by stimulation with 1 ng/mL TNFα or 10 ng/mL IL‐6 for 4 hours. Luciferase activities were determined using the Promega luciferase kits according to the manufacturer's instructions (Promega, Madison, WI, USA).

### 2.7 In vitro kinase assays

The JAK2 or IKKβ in vitro kinase assay was performed with JAK2 or IKKβ immunoprecipitates, a HTScan JAK2 (IKKβ) Kinase Assay Kit (Cell Signaling Technology, Beverly, MA, USA) and streptavidin‐coated 96‐well plates (#22351, Beaverbio, Suzhou, China). Briefly, HEK293 cells were transfected with plasmids encoding JAK2‐FLAG or IKKβ‐FLAG by Lipofectamine 2000 (Invitrogen, Carlsbad, CA, USA) for 24 hours and then lysed with 1 mL lysis buffer (50 mM HEPES [pH 7.4], 150 mM NaCl, 0.15% Triton X‐100, 0.5 mM DTT, 2 mM sodium orthovanadate [Na3VO4], 2 mM sodium fluoride [NaF], 1 mM PMSF and protease inhibitor cocktail [Sigma, 1:1000]) on ice for 30 minutes. Cell lysates were collected and centrifuged to remove the pellet. The supernatants were then immunoprecipitated with 1:50 (v/v) anti‐Flag affinity gel. The immunoprecipitates were then centrifuged and washed twice with the lysis buffer and once with the kinase reaction buffer (60 mM HEPES [pH 7.5], 5 mM MgCl_2_, 5 mM MnCl_2_, 25 μmol/L Na_3_VO_4_, 200 μmol/L ATP). JAK2 or IKKβ immunoprecipitates were incubated with DMSO or xanthatin for 30 minutes and then subjected to kinase reaction in a final volume of 50 µL kinase reaction buffer containing 1.5 μmol/L FLT3 (Tyr589) or IkB‐(Ser32) biotinylated peptide substrate at 25°C for 30 minutes. Samples were then processed according to the protocol for the HTScan JAK2 (IKKβ) Kinase Assay Kit.

### 2.8 HPLC‐MS analyses of adduct of xanthatin and GSH

About 20 μmol/L xanthatin was incubated with 1 mmol/L of GSH in 20 mM Tris‐HCl (pH 7.4) for 1 hour at 37°C. Then the sample was injected into the liquid chromatography‐mass spectrometry (LC‐MS) system using methanol/water (1:1) as the mobile phase at a rate of 0.2 mL/min.

### 2.9 Pull‐down assays

MDA‐MB‐231 cells were cultured in 100 mm culture plates to 100% confluence. The cells were then pre‐treated with Xa or DMSO for 1 hour and incubated with Alk‐Xa or DMSO for 1 hour. After that, the cells were lysed with 1 mL lysis buffer (50 mM Tris‐HCl [pH 7.4], 150 mM NaCl, 1% NP‐40, 1 mM EDTA, 1 mM PMSF and 0.1% (v/v) protease inhibitor cocktail) on ice for 30 minutes. Cell lysates were centrifuged at 12 000 *g* at 4°C for 10 minutes. 100 μmol/L biotin‐azide and freshly pre‐mixed click chemistry reaction cocktail [THPTA (2.5 mM, 500 mM stock solution in H_2_O), CuSO_4 _(0.5 mM, 100 mM stock solution in H_2_O), sodium ascorbate(2.5 mM, 500 mM stock solution in H_2_O)] was added. The mixture was incubated at room temperature for 2 hours with gentle mixing. Five volumes of pre‐chilled acetone was added and the mixture was incubated at −20°C overnight. The mixture was then centrifuged at 14 000 *g* for 10 minutes at 4°C and the pellet was washed twice with pre‐chilled acetone, air‐dried for 10 minutes and resuspended in the cell lysis buffer, followed by incubation with 5% (v/v) streptavidin agarose beads for 2 hours at room temperature. The precipitates were washed five times with the lysis buffer and dissolved in Laemmli buffer, followed by Western blot analyses.

### 2.10 In‐gel fluorescence

Overexpressed JAK2 and IKKβ proteins were immunoprecipitated as described above. The immunoprecipitates were incubated with DMSO or Alk‐Xa for 30 minutes. About 100 μmol/L rhodamine‐azide and freshly premixed click chemistry reaction cocktail described above were added. The mixtures were incubated at room temperature for 2 hours with gentle mixing. Five volumes of pre‐chilled acetone was added and the mixtures were incubated at −20°C overnight and were then centrifuged at 14 000 *g* for 10 minutes at 4°C. The pellets were air‐dried for 10 minutes and dissolved in Laemmli buffer. The proteins were resolved by 8% denaturing PAGE and the fluorescence was detected by a GE Typhoon scanner.

### 2.11 Coomassie G‐250 staining

The coomassie G‐250 staining was performed as described.^38^

### 2.12 HPLC‐MS/MS analyses of JAK2 and IKKβ

MDA‐MB‐231 cells were treated with 20 μmol/L Xa for 1 hour and lysed with lysis buffer (50 mM Tris‐HCl [pH 7.4], 150 mM NaCl, 1% NP‐40, 1 mM EDTA, 1 mM PMSF and 0.1% [v/v] protease inhibitor cocktail). The JAK2 protein was immunoprecipitated from the cell lysates with the anti‐JAK2 antibody and dissolved in Laemmli buffer. HEK293 cells were transfected with a plasmid encoding an IKKβ‐FLAG by Lipofectamine 2000 for 24 hours and lysed as described above. The IKKβ protein was immunoprecipitated by anti‐Flag affinity gel and dissolved in Laemmli buffer. Both JAK2 and IKKβ proteins were pre‐separated by SDS‐PAGE. The gel slices containing the two proteins were cut off from the PAGE and digested in gel.^39^ The drug modified peptide spectra with a Mascot ion score of more than 20 were manually inspected using stringent criteria as previously described.^40^

### 2.13 Docking analyses

Docking analyses were performed with Maestro 10.1. X‐ray co‐crystal structures of JAK2 (4Z32) and IKKB (4E3C) were downloaded from RCSB Protein Date Bank and prepared by the Protein Preparation Wizard Workflow in the Schrödinger program suite. The structure of xanthatin was prepared by the Ligand Preparation and docked into the defined binding site without constraint. Top ranking compounds were submitted and generated by Pymol based on the Prime‐score.

### 2.14 Statistical analyses

The statistical analyses were performed with the Graphpad Prism software. IC50 values were calculated by the Graphpad Prism 7. For comparisons between and within more than two groups, one‐way Analysis of Variance (ANOVA) and two‐way ANOVA were used, followed by the Dunnett's multiple comparisons test. All values are reported as mean ± *SD*.

## 3 RESULTS

### 3.1 Xanthatin inhibited the IL‐6‐induced STAT3 activation by directly inactivating JAK2 kinase activity

**Figure 1:**
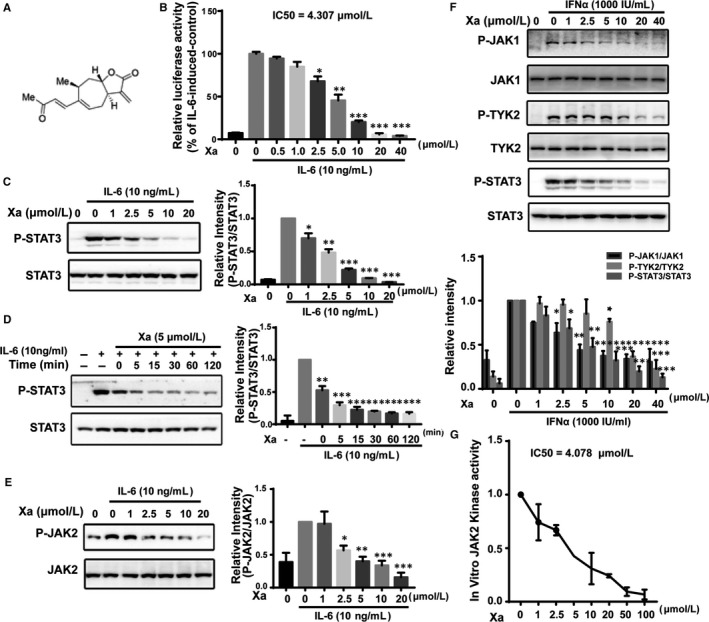
Xanthatin blocked signal transducer and activator of transcription 3 (STAT3) signalling by directly inhibiting JAK2 kinase activity. A, Structure of xanthatin. B, Effects of xanthatin on the IL‐6‐induced luciferase activity. HepG2/STAT3 cells were pre‐treated with xanthatin at indicated concentrations for 1 h and luciferase activity was measured following stimulation of IL‐6 (10 ng/mL) for 4 h. C, Xanthatin dose‐dependently inhibited IL‐6‐induced STAT3 phosphorylation. MDA‐MB‐231 cells were pre‐treated with xanthatin at various concentrations for 1 h before stimulation by IL‐6 (10 ng/mL) for 10 min. Cell lysates were processed for Western blot analysis. D, Xanthatin time‐dependently inhibited IL‐6‐induced STAT3 phosphorylation. MDA‐MB‐231 cells were incubated with 5 μmol/L xanthatin for various durations before stimulation by IL‐6 (10 ng/mL) for 10 min. Cell lysates were processed for Western blot analysis. E, Xanthatin dose‐dependently inhibited IL‐6‐induced JAK2 phosphorylation. MDA‐MB‐231 cells were pre‐treated with xanthatin at various concentrations for 1 h before stimulation by IL‐6 (10 ng/mL) for 10 min. Cell lysates were processed for Western blot analysis. F, Effects of xanthatin on the IFNα‐induced JAK1 and TYK2 phosphorylation. MDA‐MB‐231 cells were pre‐treated with xanthatin at indicated concentrations for 1 h before stimulation by IFNα (1000 IU/mL) for 10 min. Cell lysates were processed for Western blot analysis. G, JAK2 in vitro kinase assay. The overexpressed JAK2 protein immunoprecipitated from the HEK293 was subjected to in vitro kinase assay as described in Materials and Methods. (n = 3, * *P* < 0.05, **P < 0.01, ***P < 0.001 compared with IL‐6‐induced cells)

We investigated the effects of xanthatin (Figure 1A) on STAT 3 signalling using a HepG2 cell line transfected with a STAT3‐responsive luciferase reporter gene and found that the IL‐6‐induced STAT3‐responsive luciferase activity was inhibited by xanthatin in a dose‐dependent manner (IC50 = 4.307 μmol/L) (Figure 1B).

We then examined the effects of xanthatin on STAT3 tyrosine phosphorylation/activation. Xanthatin inhibited the IL‐6‐induced STAT3 phosphorylation in a dose‐ and time‐dependent manner, reaching a complete inhibition at 20 μmol/L (Figure 1C,D).

JAK2 is the major kinase of STAT3.^41^ To explore whether the inhibition on STAT3 phosphorylation was the result of inactivating JAK2 by xanthatin, we examined the effects of xanthatin on the phosphorylation/activation of JAK2. Xanthatin inhibited the IL‐6‐induced JAK2 phosphorylation in a similar fashion as that on the STAT3 phosphorylation (Figure 1E). Therefore, JAK2 appeared to be the direct target of Xa.

To investigate the specificity of xanthatin, we analysed the effects of xanthatin on the IFNα‐induced phosphorylation of JAK1 and TYK2, two other members of the JAK family. The IFNα‐induced phosphorylation of JAK1 and TYK2, as well as STAT3, was also inhibited by xanthatin (Figure 1F). It appeared that xanthatin was a pan‐JAK inhibitor.

To confirm that xanthatin is a direct inhibitor of JAK kinases, we overexpressed and immunoprecipitated JAK2 protein from HEK293 cells and performed an in vitro kinase assay. Xanthatin inhibited JAK2 kinase directly with an IC50 of 4.078 μmol/L (Figure 1G). These data suggested that xanthatin inhibited the IL‐6‐induced STAT3 activation by directly inhibiting the JAK2 kinase activity.

### 3.2 Xanthatin inhibited NF‐κB signalling by blocking IKKβ kinase activity

**Figure 2:**
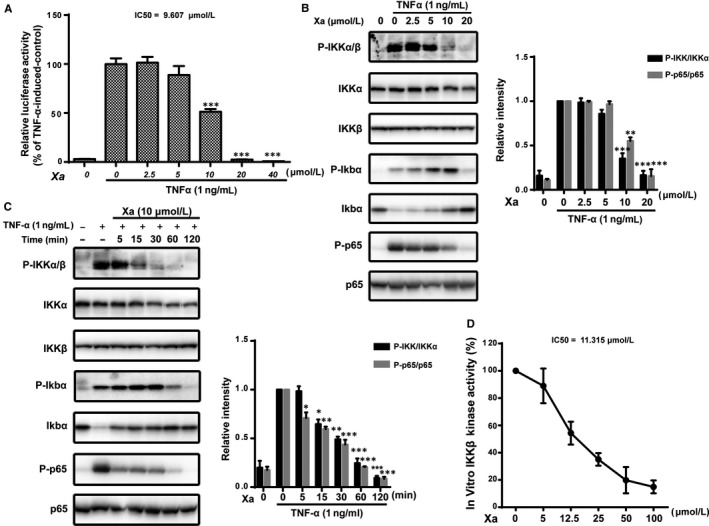
Xanthatin blocked NF‐κB signalling by directly inhibiting IKKβ kinase activity. A, Effects of xanthatin on the TNFα‐induced luciferase activity. HEK293/NF‐κB cells were pre‐treated with xanthatin at indicated concentrations for 1 h and luciferase activity was measured following stimulation of TNFα (1 ng/mL) for 4 h. B, Xanthatin dose‐dependently inhibited TNFα‐induced NF‐κB pathway. MDA‐MB‐231 cells were pre‐treated with xanthatin for 1 h before stimulation by TNFα (1 ng/mL) for 10 min. Cell lysates were processed for Western blot analysis. C, Xanthatin time‐dependently inhibited TNFα‐induced NF‐κB pathway. MDA‐MB‐231 cells were pre‐treated with 20 μmol/L xanthatin for various durations (0‐120 min) before stimulation by TNFα (1 ng/mL) for 10 min. Cell lysates were processed for Western blot analysis. D, IKKβ in vitro kinase assay. The overexpressed IKKβ protein immunoprecipitated from the HEK293 was subjected to in vitro kinase assay as described in Materials and Methods. (n = 3, *P < 0.05, **P < 0.01, ***P < 0.001 compared with TNFα‐induced cells)

We next investigated the effects of xanthatin on the NF‐κB signalling using a Hek293 cell line transfected with a NF‐κB‐responsive luciferase reporter gene and found that the TNFα‐induced NF‐κB‐responsive luciferase activity was inhibited by xanthatin in a dose‐dependent manner (IC50 = 9.607 μmol/L) (Figure 2A).

To understand the mechanisms of xanthatin in inhibiting the activation of NF‐κB, we examined the effects of xanthatin on IKKα/β. As shown in Figure 2B‐C, the TNFα‐induced phosphorylation of IKKα/β was inhibited by xanthatin in a dose‐ and time‐dependent manor, leading to the inhibition of IκBα degradation as well as p65 phosphorylation.

We then investigated the effects of xanthatin on the activity of IKKβ, the key kinase of the NF‐κB pathway, by overexpressing and immunoprecipitating the IKKβ protein from the HEK293 cells to perform an in vitro kinase assay. Xanthatin directly inhibited IKKβ kinase activity with an IC50 of 11.315 μmol/L (Figure 2D). Hence, xanthatin blocked NF‐κB signalling by directly inhibiting IKKβ kinase.

### 3.3 The activity of xanthatin was blocked by GSH and was dependent on its α‐methylene‐γ‐butyrolactone group

**Figure 3:**
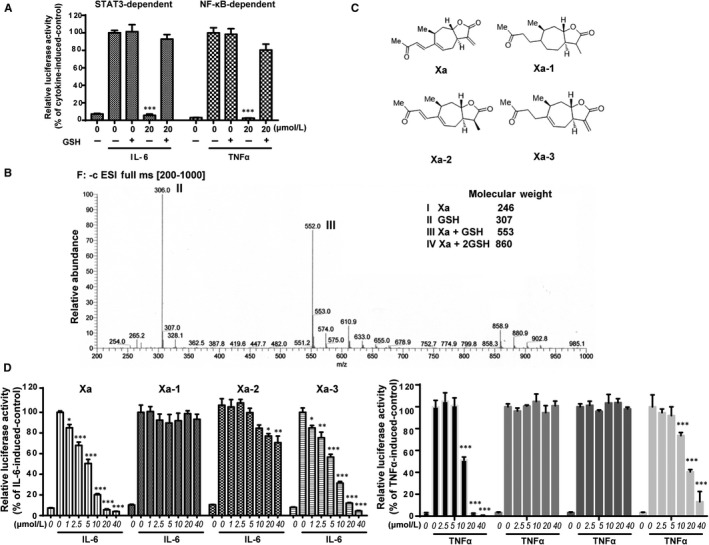
Xanthatin had the potential to react with protein thiols and its activity depended on its α‐methylene‐γ‐butyrolactone moiety. A, GSH blocking assay. HepG2/STAT3 cells were pre‐treated with 1 mmol/L GSH, xanthatin or their mixture for 1 h and then stimulated with IL‐6 for 4 h. Cells were harvested for luciferase assay. B, LC‐MS analysis of the incubation product of xanthatin and GSH. About 20 μmol/L xanthatin was incubated with 1 mmol/L GSH for 1 h at 37°C and the mixture was resolved by LC‐MS as described in Materials and Methods. Molecular weights of the molecules are indicated. C, Xanthatin and its derivatives. D, The effects of xanthatin and its derivatives on STAT3 activation were measured by luciferase assay. HepG2/STAT3 cells were pre‐treated with Xa, Xa‐1, Xa‐2 and Xa‐3 for 1 h and luciferase activity was measured following stimulation of IL‐6 (10 ng/mL) for 4 h. E, The effects of xanthatin and its derivatives on NF‐κB activation were measured by luciferase assay. HEK293/NF‐κB cells were pre‐treated with Xa, Xa‐1, Xa‐2 and Xa‐3 for 1 h and luciferase activity was measured following stimulation of TNFα (1 ng/mL) for 4 h. (n = 3, *P < 0.05, **P < 0.01, ***P < 0.001 compared with cytokine‐induced cells)

The α, β‐unsaturated carbonyl group of xanthatin can serve as a site for Michael addition, react with protein thiols and covalently bind to the proteins.^42^, ^43^ To find out whether xanthatin is a covalent inhibitor, we pre‐incubated xanthatin with the thiol‐containing glutathione (GSH) and then to examine whether this pre‐incubation could alleviate the inhibitory effects of xanthatin on the two signalling pathways. The inhibitory effects of xanthatin were completely abrogated by GSH (Figure 3A).

We next analysed the incubation products of xanthatin and GSH by LC‐MS. As xanthatin possesses two α, β‐unsaturated carbonyl groups, there should be three products if both α, β‐unsaturated carbonyl groups were reactive. However, there was only one major product with a molecular weight of 553, suggesting an addition of one molecule of GSH to one molecule of xanthatin (Figure 3B).

We then assessed the reactivity of the two α, β‐unsaturated carbonyl groups of xanthatin to understand their contributions to the inhibitory activity of xanthatin by structure modifications of the two α, β‐unsaturated carbonyl groups. As shown in Figure 3D‐E, the major α, β‐unsaturated carbonyl group that contributed to the inhibition of JAK/STAT and NF‐κB signalling pathways was the α‐methylene‐γ‐butyrolactone group. Modification of the other group had very little effects. Taking together, these data suggested that xanthatin was a covalent inhibitor and its inhibitory activity mainly relied on its α‐methylene‐γ‐butyrolactone group.

### 3.4 Xanthatin covalently modified Cys243 of JAK2 and Cys412, Cys464 of IKKβ

**Figure 4:**
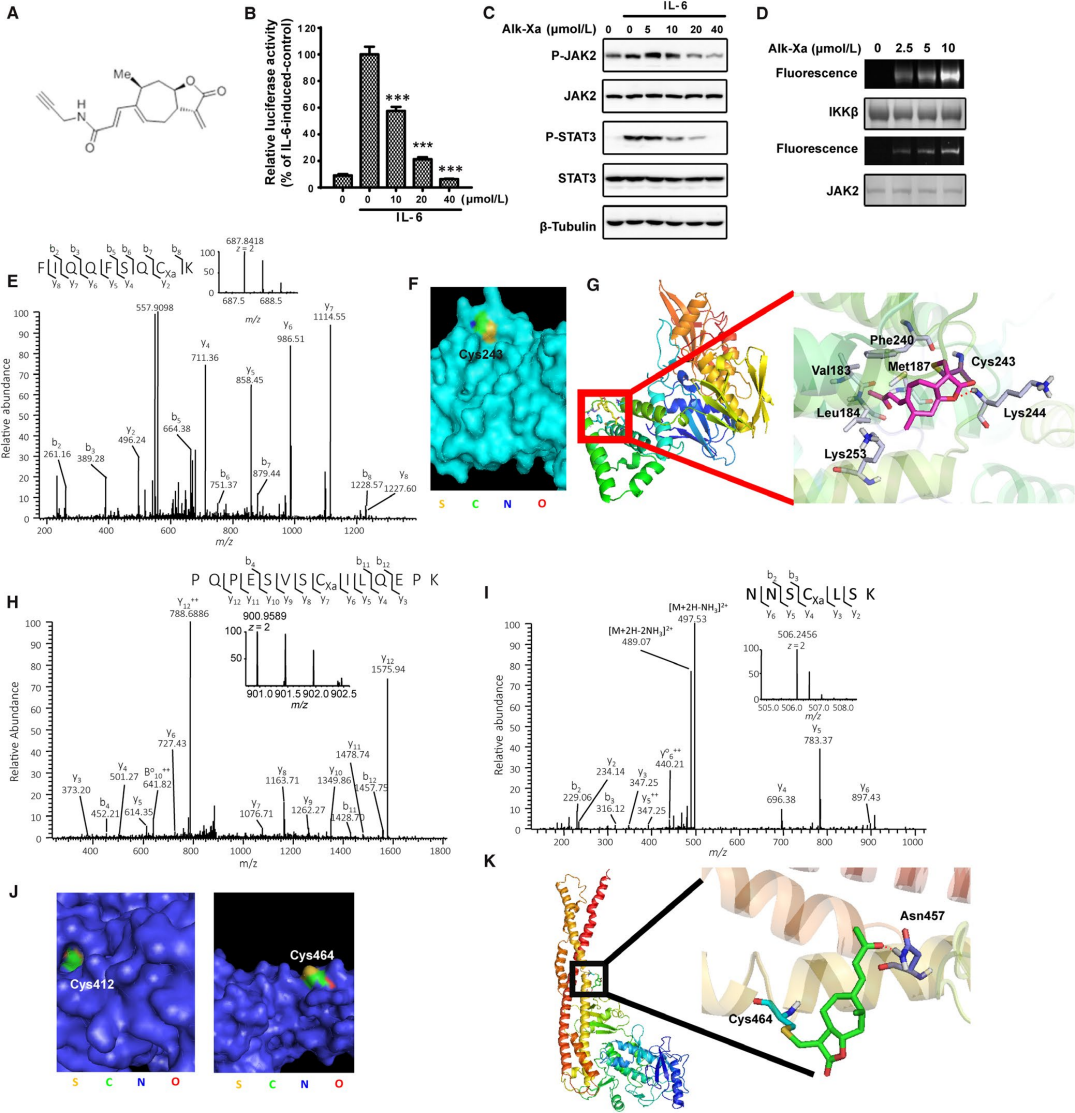
Xanthatin covalently modified Cys243 of JAK2 and Cys412, Cys464 of IKKβ. A, Structure of Alk‐Xa. B, Effects of Alk‐Xa on IL‐6‐induced luciferase activity. HepG2/STAT3 cells were pre‐treated with Alk‐Xa for 1 h and luciferase activity was measured following stimulation of IL‐6 (10 ng/mL) for 4 h. (n = 3, ***P < 0.001 compared with IL‐6‐induced cells) C, Effects of Alk‐Xa on IL‐6‐induced JAK2/STAT3 phosphorylation. MDA‐MB‐231 cells were treated with Alk‐Xa for 1 h before stimulation by IL‐6 (10 ng/mL) for 10 min. Cell lysates were processed for Western blot analysis. D, In‐gel fluorescence scanning of Alk‐Xa‐treated JAK2 and IKKβ protein. Purified JAK2 and IKKβ proteins were incubated with Alk‐Xa for 30 min and subsequently subjected to click chemistry with rhodamine‐alkyne as described in Materials and Methods. In‐gel fluorescence scanning was showed. E, Xanthatin covalently bound to Cys243 of JAK2. MDA‐MB‐231 cells were incubated with 20 μmol/L xanthatin for 1 h. After that, JAK2 protein was immunoprecipitated from cell lysate and subjected to LC‐MS/MS analysis as described in Materials and Methods. F, The position of Cys243 on JAK2 protein. The crystal structure of JAK2 (4Z32) was from RCSB and generated by Pymol. The Cys243 of JAK2 was coloured. G, A representative view of the xanthatin binding to Cys243. The docking study was performed with Schrödinger program suite as described in Materials and Methods. H, xanthatin covalently binds to Cys412 of IKKβ. HEK293 cells were transfected with IKKβ plasmid and were incubated with 20 μmol/L xanthatin for 1 h. The overexpressed IKKβ protein was immunoprecipitated from cell lysate and subjected to LC‐MS/MS analysis as described in Materials and Methods. I, xanthatin covalently bound to Cys464 of IKKβ. HEK293 cells were transfected with IKKβ plasmid and were incubated with 20 μmol/L xanthatin for 1 h. The overexpressed IKKβ protein was immunoprecipitated from cell lysate and subjected to LC‐MS/MS analysis as described in Materials and Methods. J, The position of Cys412 and Cys464 on IKKβ protein. The crystal structure of IKKβ (4E3C) was from RCSB and generated by Pymol. The Cys412 and Cys464 of IKKβ were coloured. K, A representative view of the xanthatin binding to Cys464. The docking study was performed with Schrödinger program suite as described in Materials and Methods

Above data suggested that xanthatin was a covalent inhibitor. To verify whether xanthatin covalently modified JAK2 and IKKβ, we synthesized an alkyne‐containing xanthatin analogue Alk‐Xa (Figure 4A). Alk‐Xa maintained the biological activities of xanthatin (Figure 4B‐C). We then overexpressed and immunoprecipitated the JAK2 and IKKβ proteins from HEK293 cells and incubated them with Alk‐Xa. The incubation products were subsequently subjected to click chemistry with rhodamine‐azide. The in‐gel fluorescence scanning showed that the fluorescence intensity of JAK2 and IKKβ was proportional to the dosage of Alk‐Xa, indicating that Alk‐Xa covalently bound to JAK2 and IKKβ (Figure 4D).

To identify the specific residues of JAK2 modified by xanthatin, we incubated MDA‐MB‐231 cells with xanthatin and the JAK2 proteins were immunoprecipitated and subjected to LC‐MS/MS analysis. The UniProt database searching using MASCOT algorithm (http://www.matrixscience.com/) identified a peptide with a calculated mass that was 246.30 Da larger than the cysteine‐containing peptide FIQQFSQCK. The mass difference matched the molecular weight of one xanthatin molecule, suggesting that the Cys243 of JAK2 was covalently modified by xanthatin (Figure 4E).

We then analysed the location of Cys243 in the crystal structure of JAK2.^46^ As shown in Figure 4F, Cys243 was on the surface of JAK2 protein, suggesting the accessibility of Cys243 by xanthatin.

To understand how xanthatin may interact with Cys243, we retrieved and prepared the crystal structure JAK2 (4Z32) from the Protein Data Bank (PDB) and performed a virtual docking analysis. The binding mode showed that the oxygen of lactone in xanthatin formed a stable H‐bond with the residue Lys244 of JAK2. In addition, the side chain of xanthatin extended to a hydrophobic pocket made up of Leu184, Met187, Phe240 and Val183. The H‐bond and hydrophobic‐lipophilic interactions likely formed the binding site for xanthatin (Figure 4G).

We next performed similar analysis for the interaction between xanthatin and IKKβ and found that the Cys412 and Cys464 of IKKβ were covalently modified by xanthatin (Figure 4H‐I). The locations of Cys412 and Cys464 in the crystal structure of IKKβ were also examined.^47^ We found that the Cys464 was on the surface, while the Cys412 was located deep in a cavity of IKKβ (Figure 4J). In the molecular docking study, only the Cys464 could be successfully docked and the binding mode of xanthatin to the Cys464 showed that the carbonyl group of the side chain of xanthatin formed an H‐bond with the Asn457 of IKKβ. The estimated free energy of xanthatin to cys464 was much lower, suggesting that cys464 was more easily to be attacked by xanthatin (Figure 4K).

### 3.5 Xanthatin preferentially bound to JAKs and IKKs

**Figure 5:**
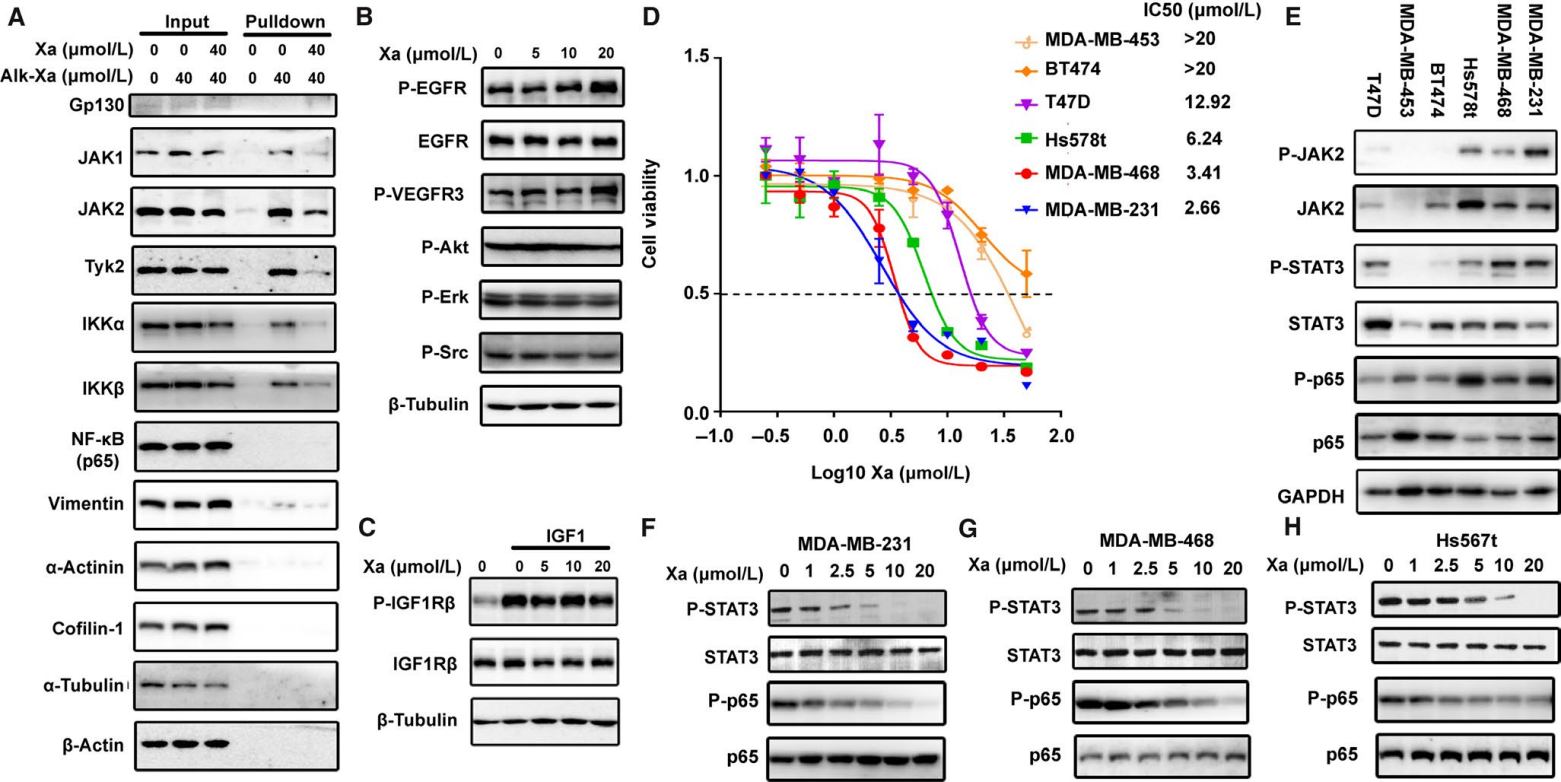
Xanthatin selectively bound to JAKs and IKKs and preferentially inhibited the growth of cancer cells with constitutively activated STAT3 and p65. A, Pull‐down assay. MDA‐MB‐231 cells were pre‐treated with xanthatin or DMSO for 1 h and were incubated with Alk‐Xa or DMSO for 1 h. Cells were lysed and subjected to bio‐orthogonal click chemistry. Streptavidin resin was used to pull down targeted proteins. The precipitates were processed for Western blot analysis. B, Effects of xanthatin on EGFR, VEGFR3, Akt, Erk and Src phosphorylation. MDA‐MB‐231 cells were incubated with xanthatin for 1 h. Whole cell lysates were subjected to Western blot analysis. C, Effects of xanthatin on the IGF1‐induced IGF1Rβ phosphorylation. MDA‐MB‐231 cells were pre‐incubated with xanthatin for 1 h and stimulated with IGF1 (50 ng/mL) for 10 min. Cell lysates were processed for Western blot analysis. D, IC50 of xanthatin on growth of different human breast cancer cell lines. Cells were treated with xanthatin for 72 h and the drug effect on cell growth was determined by MTT assay. E, The expression and activation of JAK‐STAT and NF‐κB pathway in different breast cancer cells. Cells were lysed and subjected to Western blot analysis. F‐H, Xanthatin inhibited constitutive STAT3 and p65 phosphorylation in MDA‐MB‐231, MDA‐MB‐468 and Hs578t cells. Cancer cells were treated with xanthatin for 1 h. Cell lysates were processed for Western blot analysis

Many proteins, including abundant proteins like tubulin and actin, contain exposed cysteine residues that can potentially be modified by reactive oxygen/nitrogen species and electrophiles.^48^, ^49^ To determine whether xanthatin indiscriminately reacted with cysteine‐containing proteins, we used Alk‐Xa to pull down the xanthatin‐interacting proteins. After incubation with Alk‐Xa, the cells were lysed and precipitated with streptavidin resin and subjected to Western blotting analysis. As shown in Figure 5A, JAK1, JAK2, Tyk2, IKKα and IKKβ were pulled down with the Alk‐Xa and the interactions could be competed away by excessive unlabelled xanthatin, indicating that Alk‐Xa interacted with these proteins. On the contrary, Alk‐Xa failed to pull down gp130 and p65, two components in the JAK/STAT and NF‐κB pathways respectively. We also analysed several abundant proteins, such as tubulin, actin, cofilin and α‐actinin, to examine the selectivity of xanthatin. Our data demonstrated that Alk‐Xa could not pull down these proteins even though their abundances were much higher than that of JAKs and IKKs. We also analysed possible interactions of xanthatin with six randomly chosen protein kinases and found that xanthatin did not inhibit the phosphorylation of these kinases (Figure 5B‐C).

Taken together, these data suggested that xanthatin selectively interacted with JAKs and IKKs.

### 3.6 Xanthatin preferentially inhibited the growth of cancer cells with constitutively activated STAT3 and p65

STAT3 and NF‐κB play key roles in survival and growth of malignant cells via controlling expressions of cell survival/death, proliferation and immune response genes. Therefore, we examined the effects of xanthatin on the growth of a panel of human cancer cell lines. Xanthatin induced significantly more death in MDA‐MB‐231, MDA‐MB‐468 and Hs578t cells than in other cells (Figure 5D). Because xanthatin inhibited the JAK/STAT3 and NF‐κB signalling pathways, we next examined the activation status of the signalling proteins in the two pathways in these cancer cell lines. Phosphorylation of STAT3 and p65 was higher in the MDA‐MB‐231, MDA‐MB‐468 and Hs578t cells and could be inhibited dose‐dependently by xanthatin (Figure 5F‐H). The phosphorylation of IKKβ and IκB could not be detected in these cell lines (data not shown). These data demonstrated the potential of xanthatin to selectively inhibit the growth of cancer cells through blocking the constitutively activated STAT3 and p65.

## 4 DISCUSSION

Herb plant *Xanthium L.* has been used widely in China as an anti‐inflammatory medicine and xanthatin is an abundant component of the plant. It has been reported to inhibit the STAT3 and NF‐κB signalling. However, the molecular mechanisms of the inhibition are still unknown. Here, we report that xanthatin is a covalent inhibitor of JAKs and IKKs. Xanthatin potently inhibited the IL‐6‐induced activation of STAT3 as well as the TNFα‐induced activation of NF‐κB by covalently targeting the JAKs and IKKs.

LC/MS analysis demonstrated that the Cys243 of JAK2 and the Cys412 and Cys464 of IKKβ were covalently modified by xanthatin. The Cys412 and Cys464 of IKKβ are located between the ULD motif and LZ motif of IKKβ, both of which are required for the kinase activity of IKKβ, explaining the inhibitory effects of xanthatin on the IKKβ kinase activity.^52^, ^53^ The Cys243 of JAK2 is located in the FERM domain, which is necessary for the interactions of JAKs with the cytoplasmic tails of the receptors.^46^, ^54^, ^55^The physical interactions between JAK2 and gp130, however, were not affected by xanthatin (data not shown), suggesting that xanthatin may inactivate the JAKs by changing their conformations.

Our data demonstrated that xanthatin was a selective covalent inhibitor. The human JAK2 protein contains 27 cysteines, while the IKKβ contains 18 cysteines. Xanthatin however only interacted with the Cys243 of JAK2 and the Cys412 and Cys464 of IKKβ. In addition, xanthatin did not bind abundant proteins such as tubulin and actin because they were not pulled down by the xanthatin derivative Alk‐Xa. Furthermore, xanthatin did not affect the phosphorylation of other kinases like EGFR, VEGFR3, IGF‐IR, Erk, Akt and Src.

Xanthatin preferentially inhibited the growth of the cancer cells with constitutively activated STAT3 and p65. The dual activities of xanthatin targeting both the JAK/STAT pathway and the NF‐κB pathway may render xanthatin as a more effective anticancer drug candidate over other single pathway‐targeted drugs.

## CONFLICT OF INTEREST

No potential conflicts of interest are disclosed.

## AUTHOR CONTRIBUTIONS

Qiang YU and Man LIU designed the study. Qiang YU and Man LIU drafted the manuscript. Man LIU performed and analysed the experiments shown in Figures 1‐6. Ming‐wei SUN and Min‐jia TAN conducted and analysed the HPLC–MS/MS experiments. Cheng‐qian XIAO and Li‐hong HU provided xanthatin and its derivatives. All authors analysed the results and approved the final version of the manuscript.
