# Supplementary figures and images for: Xanthatin inhibits STAT3 and NF‐κB signalling by covalently binding to JAK and IKK kinases
Source: J Cell Mol Med. 2019 Apr 16;23(6):4301–12. doi: 10.1111/jcmm.14322 (PMC6533482; doi:10.1111/jcmm.14322)

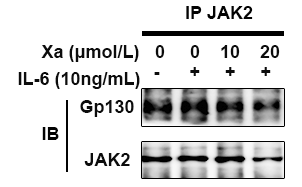

Supplement: Supplementary file 1 [file JCMM-23-4301-s001.tif]
